# Higher Iron Intake Is Independently Associated with Obesity in Younger Japanese Type-2 Diabetes Mellitus Patients

**DOI:** 10.3390/nu14010211

**Published:** 2022-01-04

**Authors:** Efrem d’Ávila Ferreira, Mariko Hatta, Yasunaga Takeda, Chika Horikawa, Mizuki Takeuchi, Noriko Kato, Hiroki Yokoyama, Yoshio Kurihara, Koichi Iwasaki, Kazuya Fujihara, Hiroshi Maegawa, Hirohito Sone

**Affiliations:** 1 Department of Hematology, Endocrinology and Metabolism, Faculty Medicine, Niigata University, 1-757 Asahimachi-dori, Chuoh-ku, Niigata 951-8510, Japan; efremdavila@gmail.com (E.d.F.); hatta@ngt.saiseikai.or.jp (M.H.); yasutake@adm.niigata-u.ac.jp (Y.T.); horikawa@unii.ac.jp (C.H.); takeuchi@nuhw.ac.jp (M.T.); kafujihara-dm@umin.ac.jp (K.F.); 2 Department of Health and Nutrition, Faculty of Human Life Studies, University of Niigata Prefecture, 471 Ebigase, Higashi-ku, Niigata 950-8680, Japan; 3 Department of Health and Nutrition, Niigata University of Health and Welfare, 1398 Shimami-cho, Kita-ku, Niigata 950-3198, Japan; 4 Kato Clinic of Internal Medicine, 201, 3-11-14 Talasago, Katsushika-ku, Tokyo 125-0054, Japan; norikokato.0131@gmail.com; 5 Jiyugaoka Medical Clinic, 6-4-3 Nishirokujominami, Obihiro 080-0016, Japan; dryokoyama@yokoyamanaika.com; 6 Kurihara Clinic, 5-7-28 Atsubetsuchuosanjo, Sapporo Atsubetsu-ku, Sapporo 004-0053, Japan; ykuri@yg7.so-net.ne.jp; 7 Iwasaki Naika Clinic, 1-30-13 Minamiiwakunimachi, Iwakuni 740-0034, Japan; kiwasaki@themis.ocn.ne.jp; 8 Department of Medicine, Division of Diabetology, Endocrinology and Nephrology, Shiga University of Medical Science, Seta Tsukonowa-cho, Otsu 520-2192, Japan; maegawa@belle.shiga-med.ac.jp

**Keywords:** iron, dietary intake, obesity

## Abstract

We aimed to analyze the association between dietary iron intake and obesity assessed by BMI after adjustment for nutrient intake (macronutrients and fiber) and food groups. The study design was cross-sectional. Patients with type-2 diabetes (*n* = 1567; 63.1% males; mean age 62.3 ± 11.6 years) were included in the study. To assess diet, consumption of typical food groups was determined by a food frequency questionnaire. Obesity was defined as BMI ≥ 25 kg/m^2^. We performed a binary regression analysis between quartiles of iron intake and obesity by quartiles of age group. A direct linear association was found for the highest quartile of iron intake and obesity in the younger age group of 30 to 54 years (OR = 3.641, 95% CI = 1.020–12.990; *p* trend = 0.011). Multivariate analysis using food groups as opposed to nutrients revealed a positive trend for obesity in the younger age group after adjusting for lifestyle factors, energy intake and bean and vegetable intake (*p* trend = 0.023). In all participants, an inverse association was observed before adjustment by vegetable intake (OR = 0.453, 95% CI = 0.300–0.684; *p* trend = 0.001). Higher iron intake was associated with obesity independent of macronutrient and fiber intake but only in the youngest quartile of age group examined.

## 1. Introduction

Obesity is the main modifiable risk factor for type-2 diabetes, with approximately 80% to 85% of people with this disease being overweight or obese [1]. Despite their higher dietary intake, micronutrient deficiencies have been reported in people with obesity [2]. According to a quantitative metanalysis, a significant correlation between iron deficiency and obesity was reported in studies that did not include a ferritin-based diagnosis, but this was not seen when studies with ferritin-based diagnosis were reviewed [3]. Thus, ferritin was found to be positively associated with obesity; however, it is a marker of inflammation rather than iron status in overweight or obese individuals. Iron deficiency in people with obesity may be a consequence of multiple factors including low iron absorption, iron sequestration as a result of the chronic inflammation resulting from obesity, physical inactivity or increased blood volume [3].

On the other hand, excess iron levels are deleterious and can generate reactive oxygen species leading to DNA damage and lipid peroxidation. In turn, these factors are associated with cardiovascular disease, type-2 diabetes and cancer [4]. Studies that assessed dietary iron intake and health outcomes have reported that a higher iron intake was associated with increased prevalence of metabolic syndrome or some of its components [4,5,6,7]. However, regarding studies assessing obesity as an outcome, disparate results have been reported, with some finding that higher iron intake was protective against obesity [8,9] and others finding no association [10,11,12]. Insufficient adjustment for nutritional confounding factors might explain the current divergence between such studies. Furthermore, correlations between iron and food groups were not characterized in previous obesity studies, which could also help to explain the diverging results.

Therefore, we aimed to analyze associations between dietary iron intake and obesity as measured by BMI after adjustment for nutrient intake (macronutrients and fiber) and food groups in people with type-2 diabetes from across Japan.

## 2. Materials and Methods

### 2.1. Study Population and Design

This was a cross-sectional study using a database of patients with type-2 diabetes from clinics across Japan participating in the Japan Diabetes Clinical Data Management Study Group (JDDM), which was collected from the year 2000 to 2002. Details on the data collection software and the JDDM are described elsewhere [13]. The cohort consisted of 1567 Japanese type-2 diabetes patients aged 30 to 89 years, with 988 (63.1%) being male.

### 2.2. Dietary and Physical Assessment

The diet was assessed by a validated Food Frequency Questionnaire (FFQg), which was self-administered [14]. For each food group, a standard/normal amount of representative foods was shown and four options were given for morning, lunch and dinner: Do not eat, A little, Normal and Plenty. Number of times per week of the typical intake for morning, lunch and dinner was also queried. Standardized software was used to calculate the nutrient intake (Eiyo-kun; Kenpakusha Co., Ltd., Tokyo, Japan) [15]. Supplement intake was not incorporated into the nutritional intake. Regarding the physical assessment, height and weight were self-reported by the participants. Physical activity was calculated using a Japanese version of the International Physical Activity Questionnaire (IPAQ), short form [16,17], which was self-administered by the participants. Smoking status was assessed at the time of the nutritional questionnaire.

### 2.3. Statistical Analysis

Categorical variables were expressed as numerals and percentages, and were compared by Chi Square testing. Continuous variables were expressed as means and standard deviations and were compared by the Jonckheere test. Binary logistic regression analysis was used to assess iron intake and obesity, defined as BMI ≥ 25 kg/m^2^ according to the Japan Society for the Study of Obesity [18]. Age and iron intake were divided into quartiles and correlations between iron intake and food groups were calculated by r^2^. Lifestyle and nutritional factors were included as potential confounders. All statistical analyses were performed using SPSS software version 27.0 (IBM Corp., Armonk, NY, USA) and a significant difference was defined as *p* < 0.05, while the confidence interval was set at 95%.

### 2.4. Ethical Considerations

The procedures of the study were in accordance with the ethical standards of the JDDM, Niigata University and Health Research Involving Human Subjects in Japan, and with the Helsinki Declaration or comparable ethical standards. Informed consent was obtained from all participants included in the study.

## 3. Results

### 3.1. Characteristics of the Study Participants

The characteristics of the study participants are described in Table 1. The mean age was 62.3 (SD 11.6) years and the mean iron intake of men and women was 7.0 mg/d and 6.9 mg/d, respectively (data not shown). Except for energy and saturated fat intake, there were several significant differences between age groups regarding food groups and nutrients. Vegetables, beans, seafood and fruit intakes increased with age, while meat intake decreased with age. Higher BMI, higher prevalence of current smoking and drinking and the lowest amount of exercise characterized members of the younger age group (quartiles of age were 30–54, 55–63, 64–71 and 72–89 years). Iron intake quartiles were ≤5.7, 5.8–6.9, 7.0–8.3 and ≥8.4 mg/d and the first quartile was used as a reference.

### 3.2. Correlations between Intake of Iron and Food Groups

Supplementary Table S1 shows the correlations between iron and food groups in the whole cohort. The food groups of which the intake was most closely correlated with iron were beans (0.422), vegetables (0.372) and seafood (0.251).

### 3.3. Multivariate Analysis of Associations between Iron Intake and Obesity Adjusted by Nutrient Intake

Table 2 shows the multivariate analysis of associations between quartiles of iron intake and obesity by quartile of age, using models adjusted by nutrients (macronutrients and fiber). No association of iron intake and obesity was discernible in the sex-adjusted analysis (Model 1). Adjusting for lifestyle factors, energy intake and macronutrients resulted in a close-to-significant *p* trend in the youngest group (OR = 1.482, 95% CI = 0.514–4.272; *p* trend = 0.060) (Model 2). Including total fiber in the model resulted in a direct linear association for the highest quartile of iron intake and obesity in members of the younger age group (OR = 3.641, 95% CI = 1.020–12.990; *p* trend = 0.011) (Model 3).

### 3.4. Correlations between Intake of Fiber and Food Groups

Supplementary Table S2 shows the correlations between iron and food groups for all participants. The food groups most closely associated with fiber intake were vegetables (0.662), fruits (0.263) and beans (0.254).

### 3.5. Multivariate Analysis of Associations between Iron Intake and Obesity Adjusted by Food Group

Table 3 used models adjusted by food group instead of by nutrients. We adjusted by the food groups that were sources of both iron and fiber. When analyzing all participants, there was a strong protective association between higher iron intake and obesity, which disappeared after adjusting by the vegetable food group (Model 2) but was maintained after adjustment by beans (Model 3). Compared to Model 1, which was adjusted by lifestyle factors and energy intake as the only nutritional factor, the models with adjustment by beans and vegetables showed an increase in the obesity risk at the higher quartiles of iron intake by members of the youngest age group. After adjusting for both beans and vegetables, a significant *p* trend was found only in the youngest age group (*p* trend = 0.023) (Model 4).

### 3.6. Relationships between Intake of Iron and Food Groups

**Figure 1:**
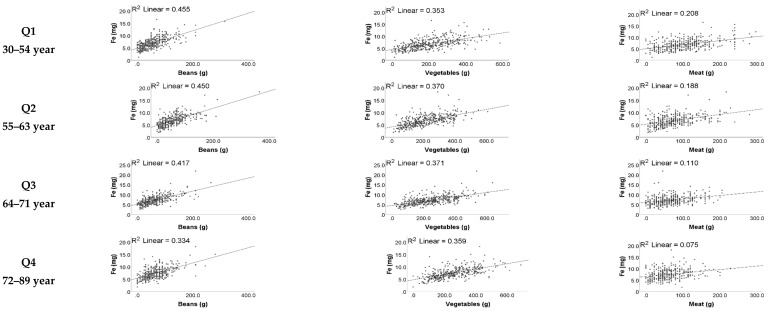
Scatter plot of Fe intake by food group according to age quartile.

Figure 1 shows correlations of iron intake and food groups by age. Meat intake had a higher correlation with iron for the first quartile of age group (0.208) and a lower correlation in the older group (0.075). For iron intake and vegetable consumption, a higher correlation was seen for the second quartile of age group (0.415) and a lower correlation was observed in the younger group (0.366). Regarding iron intake and beans, there was a higher correlation in the younger age group (0.455) and a lower correlation in the older group (0.334).

### 3.7. Multivariate Analysis of Associations between Meat Intake and Obesity

Supplementary Table S3 shows the multivariate analysis between quartiles of meat intake and obesity (BMI 25 kg/m^2^) in the quartiles of age groups adjusted by energy and fiber but not nutrients, since the variable of interest was a food group. Meat intake quartiles were ≤45.7, 48.6–68.6, 71.4–102.9 and ≥105.7 g/d and the first quartile was used as a reference. No association was observed after adjustment for all confounders.

### 3.8. Stratified Multivariate Analysis of Associations between Iron Intake and Obesity Adjusted by Nutrient Intake

Supplementary Table S4 includes further multivariate analysis on two wider age groups (≤65 and >65 years old) and gender. After adjusting for all confounders, no association was found using these categorizations.

### 3.9. Multivariate Analysis of Associations between Iron Intake and Overweight and Obesity According to World Health Organization (WHO) Definitions Adjusted by Nutrient Intake

Supplementary Table S5 shows the multivariate analysis between quartiles of iron intake and the World Health Organization (WHO) BMI cut-off points of overweight (23 kg/m^2^) and obesity (27.5 kg/m^2^) for the Asian population [19]. The WHO obesity cut-off was sensitive enough to detect a positive *p*-trend for iron intake in the whole population (*p* trend = 0.026) and in the younger quartile of age group (*p* trend = 0.017); however, no significant association was found.

## 4. Discussion

Our analysis of 1567 Japanese type-2 diabetes patients revealed that those in the highest quartile of iron intake (≥8.4 mg/d) had a significantly increased risk of obesity only in the youngest quartile of age group (30–54 years). This was after adjusting by lifestyle and nutritional factors including macronutrients and fiber intake. In the multivariate analysis by food group, iron intake from beans and vegetables was shown to have a protective effect against obesity, in that adjusting by these food groups revealed a tendency for increased obesity risk in the youngest age group as well as a loss of the inverse associations in the other age groups.

Members of the youngest age group had a comparatively high consumption of meat and the adverse effect on obesity of iron from meat intake may have been balanced by the protective effect of the fiber-rich food groups (beans and vegetables). To investigate this hypothesis, we performed a further multivariate analysis assessing the effect of meat intake on obesity (Supplementary Table S3), but no significant association was observed. It is possible that, due to the indirect nature of the dietary assessment, micronutrient interactions may have affected the observation. Mechanistically, red meat intake is correlated with an increase in ferritin levels, an iron store marker, which, in turn, was found to be associated with oxidative stress, hepatic damage and insulin resistance [20]. Because the meat food group comprised both red and processed meat in our study, it was not possible to determine whether one or the other mainly contributed to the effect on BMI. Meat is a rich source of highly available iron, but on the other hand, it also has a high fat content.

Contrary to our findings, some studies reported that plant sources of iron increased metabolic syndrome or some of its components, which can be explained by differences in diets among diverse populations [4,6,21]. In China, a higher intake of plant sources of iron was found to confer a greater risk for metabolic syndrome, including obesity as measured by waist circumference [6]. Grains and potatoes were the main sources of iron in that study. One explanation for this result is that the grains or potatoes may have been refined, a process that removes fiber and other nutrients that are protective against obesity. In Brazil, the quintile groups in which plant sources of iron intake were found to be directly associated with hyperglycemia also had the lowest intake of beans, suggesting a protective effect of this food source, probably due to its high fiber content [4].

The bean food group in our study was represented mainly by soy products including tofu and natto. Tofu, also known as bean curd, is made by curdling soymilk and then pressing the curds into blocks. It provides the required amino acids, along with minerals such as iron, calcium and magnesium, vitamins and omega-3 fatty acids. Natto is made of fermented soybeans and is rich in iron and functional compounds such as fiber, probiotics, isoflavone and a special enzyme called nattokinase; its intake was found to be associated with lower cardiovascular disease mortality [22]. Despite the presence of oxalate and phytate, two compounds that inhibit mineral absorption, the bioavailability of iron in soybeans is quite high [23].

The vegetable food group had the second highest correlation with iron intake in our study subjects, with green and yellow vegetables such as broccoli and spinach known to be rich sources of iron in this food group. Compared with soybeans, there is a lower bioavailability of iron from green and yellow vegetables [24]. Iron absorption from plant sources is lower than from animal sources, but it can be improved by simultaneously consuming foods rich in vitamin C, as well as avoiding the intake of coffee, tea or calcium in conjunction with iron-rich plant foods [25]. However, in people with obesity, iron absorption as well as the enhancing effect of vitamin C are reduced [26]. Therefore, preventing obesity could be a dietary strategy that can improve iron status in the population.

The main strengths of this study include the adjustment for lifestyle covariates and nutritional factors including macronutrient and fiber intake. Moreover, we also performed an analysis that was adjusted by individual food group, which enabled us to observe the effect of iron intake separately. Age stratification analysis was also an advantage of this study, considering that iron intakes and sources varied significantly with age. Finally, our sample of 1576 subjects of both sexes from across the country, and with a wide age range, makes the results more statistically precise and representative of the entire population. The limitations of this study include a lack of temporal relationships due to its cross-sectional nature, no direct measure of nutritional intakes and of intercorrelations between iron and other micronutrients, and no biochemical data on iron status or body composition.

In conclusion, we found that higher iron intake was independently associated with obesity in younger Japanese type-2 diabetes patients. Fiber was an important confounder of the association between iron and obesity considering that the main sources of iron in this population came from beans and vegetables. After adjusting by total fiber intake, a direct association was found for the youngest quartile of age, which was not explained by the higher meat intake in this group. In the adjusted analysis by food group using all participants’ data, higher iron intake was inversely associated with obesity before adjustment by vegetable intake. Finally, dietary iron sources varied significantly by age and this variable should be considered when assessing such associations.

## Figures and Tables

**Table 1:** Participants’ characteristics according to quartiles of age groups.

	All Participants *n* = 1567	30–54 Year *n* = 401	55–63 Year *n* = 396	64–71 Year *n* = 405	72–89 Year *n* = 365	*p* Trend
Age (years)	62.3 (11.6)	46.9 (5.5)	59.3 (2.6)	67.01 (2.2)	77.20 (4.0)	0.000
Sex (Men, %)	988 (63.1%)	291 (72.6%)	252 (63.6%)	249 (61.5%)	196 (53.7%)	<0.001
BMI (kg/m^2^)	25.8 (4.6)	28.3 (5.2)	25.9 (4.6)	24.7 (3.8)	24.4 (3.5)	0.000
Diabetes duration (years)	11.8 (7.8)	8.3 (6.1)	10.5 (6.5)	13.2 (7.6)	15.4 (8.8)	<0.001
METs (hour/week)	29.2 (39.6)	25.3 (39.4)	28.9 (39.8)	32.6 (39.6)	30.0 (39.3)	0.001
Current smoking (%)	303 (19.3%)	126 (31.4%)	90 (22.7%)	53 (13.1%)	34 (9.3%)	<0.001
Current drinking (%)	732 (46.7%)	203 (50.6%)	193 (48.7%)	201 (49.6%)	135 (37%)	<0.001
Current insulin treatment (%)	451 (28.8%)	98 (24.4%)	106 (26.8%)	105 (25.9%)	142 (38.9%)	<0.001
Current OHA and/or GLP treatment (%)	1279 (81.6%)	328 (81.8%)	328 (82.8%)	342 (84.4%)	281 (77%)	0.051
Energy (kcal/day)	1764.1 (439.1)	1807.5 (453.5)	1744.7 (456.5)	1735.2 (416.4)	1769.7 (425.8)	0.276
Protein (g/day)	65.1 (18.6)	65.3 (19.5)	63.2 (19.5)	65.0 (18.2)	67.1 (17.0)	0.036
Fat (g/day)	58.3 (19.7)	64.3 (21.9)	57.1 (19.9)	54.8 (17.4)	56.8 (17.7)	<0.001
Saturated fat (g/day)	17.9 (6.6)	19.8 (7.5)	17.4 (6.5)	16.9 (6.1)	17.4 (5.9)	0.823
Carbohydrate (g/day)	229.2 (59.4)	225.3 (60.6)	226.9 (60.9)	229.5 (54.9)	235.7 (61.1)	0.006
Fe intake (mg/day)	7.0 (2.1)	6.7 (2.2)	6.6 (2.1)	7.1 (2.1)	7.4 (2.1)	<0.001
Na intake (mg/day)	3387.0 (1252.2)	3080.8 (1147.0)	3246.6 (1154.1)	3471.7 (1212.6)	3781.5 (1390.8)	<0.001
Soluble fiber (g/day)	2.9 (1.0)	2.6 (0.9)	2.8 (0.9)	3.0 (0.9)	3.1 (1.0)	<0.001
Insoluble fiber (g/day)	9.0 (2.9)	8.2 (2.7)	8.6 (2.8)	9.5 (2.9)	9.9 (3.1)	0.000
Beans (g/day)	60.4 (42.1)	55.3 (42.1)	56.8 (41.9)	62.5 (42.6)	67.5 (40.7)	<0.001
Vegetables (g/day)	230.5 (118.2)	204.9 (114.3)	218.6 (111.2)	245.5 (121.4)	255.0 (119.4)	<0.001
Fruits (g/day)	84.1 (71.3)	49.6 (57.3)	78.22 (67.6)	101.7 (73.4)	108.7 (71.1)	0.000
Meat (g/day)	75.9 (49.6)	101.7 (56.9)	74.6 (47.8)	64.8 (41.4)	61.1 (39.2)	0.000
Seafood (g/day)	72.8 (45.3)	59.6 (42.8)	68.8 (44.3)	80.0 (44.9)	83.7 (45.4)	0.000

BMI, body mass index; METs, metabolic equivalents of tasks; OHA: oral antihyperglycemic agents; GLP: glucagon-like peptide. Data are presented as mean (standard deviation) and *n* (%).

**Table 2:** Binary regression analysis of quartiles of Fe intake and obesity by quartiles of age group adjusted by nutrients.

Age Group	Fe Intake (Quartile)	Model 1	Model 2	Model 3
		OR (CI)	OR (CI)	OR (CI)
All participants	Q1 (low)	Reference	Reference	Reference
	Q2	0.788 (0.593–1.047)	0.643 (0.485–0.992)	0.828 (0.570–1.205)
	Q3	0.909 (0.681–1.214)	0.782 (0.518–1.180)	1.080 (0.685–1.704)
	Q4 (high)	0.805 (0.606–1.071)	0.726 (0.432–1.219)	1.270 (0.681–2.366)
	*p* trend	0.315	0.259	0.233
30–54 years	Q1 (low)	Reference	Reference	Reference
	Q2	0.607 (0.347–1.063)	0.597 (0.310–1.151)	0.813 (0.405–1.631)
	Q3	1.090 (0.573–2.076)	1.279 (0.557–2.936)	2.277 (0.888–5.840)
	Q4 (high)	1.194 (0.632–2.258)	1.482 (0.514–4.272)	3.641 (1.020–12.990)
	*p* trend	0.124	0.060	0.011
55–63 years	Q1 (low)	Reference	Reference	Reference
	Q2	0.916 (0.529–1.586)	0.788 (0.411–1.512)	0.904 (0.456–1.790)
	Q3	0.901 (0.527–1.514)	0.623 (0.300–1.294)	0.790 (0.354–1.764)
	Q4 (high)	0.718 (0.414–1.245)	0.429 (0.164–1.124)	0.620 (0.207–1.854)
	*p* trend	0.703	0.377	0.847
64–71 years	Q1 (low)	Reference	Reference	Reference
	Q2	0.656 (0.378–1.139)	0.523 (0.278–0.981)	0.605 (0.308–1.190)
	Q3	0.827 (0.468–1.462)	0.524 (0.248–1.107)	0.685 (0.299–1.566)
	Q4 (high)	0.778 (0.447–1.355)	0.493 (0.195–1.244)	0.801 (0.261–2.457)
	*p* trend	0.515	0.218	0.408
72–89 years	Q1 (low)	Reference	Reference	Reference
	Q2	1.174 (0.629–2.194)	1.080 (0.535–2.182)	1.281 (0.617–2.662)
	Q3	0.918 (0.503–1.675)	0.823 (0.383–1.766)	1.141 (0.487–2.673)
	Q4 (high)	0.710 (0.390–1.291)	0.615 (0.233–1.625)	1.121 (0.348–3.614)
	*p* trend	0.406	0.574	0.913

OR, odds ratio; CI, confidence interval. Model 1: adjusted for sex and age (except in the quartiles of age group analysis). Model 2: adjusted by Model 1 plus diabetes duration, current smoking, current drinking, current insulin treatment, current OHA or GLP treatment, physical activity (METs), energy and macronutrients (fat, protein and carbohydrate). Model 3: adjusted for Model 2 plus total fiber.

**Table 3:** Binary regression analysis of quartiles of Fe intake and obesity by quartiles of age group adjusted by food group.

Age Group	Fe Intake (Quartile)	Model 1	Model 2	Model 3	Model 4
		OR (CI)	OR (CI)	OR (CI)	OR (CI)
All participants	Q1 (low)	Reference	Reference	Reference	Reference
	Q2	0.613 (0.450–0.834)	0.742 (0.537–1.025)	0.638 (0.465–0.874)	0.776 (0.558–1.079)
	Q3	0.602 (0.428–0.847)	0.837 (0.574–1.220)	0.654 (0.454–0.941)	0.916 (0.615–1.365)
	Q4 (high)	0.453 (0.300–0.684)	0.751 (0.467–1.209)	0.531 (0.330–0.854)	0.892 (0.522–1.524)
	*p* trend	0.001	0.337	0.024	0.428
30–54 years	Q1 (low)	Reference	Reference	Reference	Reference
	Q2	0.489 (0.262–0.913)	0.577 (0.300–1.109)	0.583 (0.306–1.110)	0.679 (0.348–1.327)
	Q3	0.830 (0.391–1.762)	1.152 (0.496–2.676)	1.224 (0.533–2.809)	1.651 (0.669–4.075)
	Q4 (high)	0.809 (0.336–1.946)	1.246 (0.454–3.420)	1.686 (0.560–5.074)	2.528 (0.755–8.463)
	*p* trend	0.104	0.088	0.042	0.023
55–63 years	Q1 (low)	Reference	Reference	Reference	Reference
	Q2	0.781 (0.424–1.438)	1.030 (0.537–1.978)	0.765 (0.411–1.424)	1.018 (0.524–1.977)
	Q3	0.577 (0.294–1.133)	0.867 (0.411–1.830)	0.552 (0.270–1.131)	0.846 (0.384–1.864)
	Q4 (high)	0.357 (0.156–0.817)	0.648 (0.251–1.674)	0.329 (0.128–0.844)	0.618 (0.213–1.795)
	*p* trend	0.102	0.722	0.137	0.736
64–71 years	Q1 (low)	Reference	Reference	Reference	Reference
	Q2	0.519 (0.281–0.956)	0.659 (0.346–1.258)	0.557 (0.300–1.034)	0.722 (0.374–1.395)
	Q3	0.491 (0.244–0.989)	0.707 (0.328–1.522)	0.593 (0.283–1.245)	0.886 (0.391–2.008)
	Q4 (high)	0.437 (0.194–0.986)	0.837 (0.315–2.220)	0.630 (0.246–1.612)	1.286 (0.423–3.907)
	*p* trend	0.132	0.532	0.294	0.384
72–89 years	Q1 (low)	Reference	Reference	Reference	Reference
	Q2	0.938 (0.473–1.862)	1.054 (0.522–2.127)	0.977 (0.484–1.975)	1.099 (0.535–2.257)
	Q3	0.619 (0.303–1.266)	0.789 (0.364–1.712)	0.657 (0.310–1.392)	0.838 (0.374–1.879)
	Q4 (high)	0.423 (0.174–1.030)	0.613 (0.226–1.659)	0.476 (0.175–1.290)	0.689 (0.232–2.051)
	*p* trend	0.162	0.615	0.328	0.743

CI, confidence interval; OR, odds ratio. Model 1: adjusted for sex, age (except in the quartiles of age group analysis), diabetes duration, current smoking, current drinking, current insulin treatment, current OHA or GLP treatment, physical activity (METs) and energy intake. Model 2: Model 1 and vegetables. Model 3: Model 1 and beans. Model 4: Model 1 and beans and vegetables.

## Data Availability

We are unable to provide an anonymized dataset containing the underlying data used to create the figures and tables because these data are the private property of the JDDM Study Group. Making it available to anyone in the general public would cause loss of ownership of the data by the JDDM Study Group.

## Supplementary Materials

The following supporting information can be downloaded at: https://www.mdpi.com/article/10.3390/nu14010211/s1, Table S1: Correlation coefficients between total iron intake and food groups; Table S2: Correlation coefficients between total fiber intake and food groups; Table S3: Binary regression analysis of quartiles of meat intake and obesity; Table S4: Stratified binary regression analysis of quartiles of Fe intake and obesity adjusted by nutrients; Table S5: Binary regression analysis of quartiles of Fe intake and the World Health Organization (WHO) BMI-cut-off for overweight (23 kg/m^2^) and obesity (27.5 kg/m^2^) for Asian population adjusted by nutrients.
