# Supplementary material for: Higher Iron Intake Is Independently Associated with Obesity in Younger Japanese Type-2 Diabetes Mellitus Patients
Source: Nutrients. 2022 Jan 4;14(1):211. doi: 10.3390/nu14010211 (PMC8747092; doi:10.3390/nu14010211)
Supplement: Supplementary file 1 [file nutrients-14-00211-s001.zip › Supplementary Table S1.pdf]

Supplementary Table S1.

Correlation coefficients between total iron intake and food groups

Food group	Correlation (R^2)
Beans	.422
Vegetables	.372
Seafood	.251
