# Supplementary material for: Higher Iron Intake Is Independently Associated with Obesity in Younger Japanese Type-2 Diabetes Mellitus Patients
Source: Nutrients. 2022 Jan 4;14(1):211. doi: 10.3390/nu14010211 (PMC8747092; doi:10.3390/nu14010211)
Supplement: Supplementary file 1 [file nutrients-14-00211-s001.zip › Supplementary Table S2.pdf]

**Supplementary Table S2.**

Correlation coefficients between total fiber intake and food groups

| Food group | Correlation ($R^2$) |
|---|---|
| Vegetables | .662 |
| Fruits | .263 |
| Beans | .254 |
