# Supplementary material for: Higher Iron Intake Is Independently Associated with Obesity in Younger Japanese Type-2 Diabetes Mellitus Patients
Source: Nutrients. 2022 Jan 4;14(1):211. doi: 10.3390/nu14010211 (PMC8747092; doi:10.3390/nu14010211)
Supplement: Supplementary file 1 [file nutrients-14-00211-s001.zip › Supplementary Table S3.pdf]

Supplementary Table S3. Binary regression analysis of quartiles of meat intake and obesity.

Age group	Fe intake (quartile)	Model 1<br>OR (CI)	Model 2<br>OR (CI)	Model 3<br>OR (CI)
All participants	Q1 (low)	Reference	Reference	Reference
	Q2	1.085 (.813-1.448)	1.005 (.748-1.351)	.984 (.730-1.327)
	Q3	1.035 (.783-1.368)	.917 (.682-1.232)	.885 (.656-1.193)
	Q4 (high)	1.256 (.941-1.676)	1.024 (.734-1.427)	.902 (.641-1.268)
	P trend	.462	.899	.850
30-54	Q1 (low)	Reference	Reference	Reference
	Q2	1.012 (.486-2.104)	.994 (.467-2.119)	1.024 (.477-2.198)
	Q3	1.624 (.816-3.233)	1.497 (.728-3.077)	1.591 (.768-3.297)
	Q4 (high)	1.280 (.710-2.307)	1.026 (.514-2.046)	.998 (.495-2.009)
	P trend	.477	.600	.480
55-63	Q1 (low)	Reference	Reference	Reference
	Q2	1.382 (.781-2.447)	1.298 (.718-2.346)	1.365 (.747-2.495)
	Q3	.916 (.547-1.532)	.850 (.490-1.476)	.908 (.518-1.590)
	Q4 (high)	1.145 (.652-2.010)	.929 (.486-1.778)	.899 (.465-1.738)
	P trend	.567	.605	.581
64-71	Q1 (low)	Reference	Reference	Reference
	Q2	1.191 (.697-2.036)	1.054 (.603-1.839)	1.047 (.596-1.839)
	Q3	1.097 (.652-1.847)	1.011 (.576-1.774)	.903 (.509-1.604)
	Q4 (high)	1.974 (1.095-3.557)	1.623 (.805-3.274)	1.361 (.658-2.814)
	P trend	.153	.534	.725
72-89	Q1 (low)	Reference	Reference	Reference
	Q2	.814 (.476-1.394)	.799 (.453-1.407)	.719 (.403-1.282)
	Q3	.829 (.461-1.491)	.717 (.371-1.383)	.654 (.335-1.277)
	Q4 (high)	.895 (.458-1.750)	.724 (.345-1.517)	.557 (.256-1.213)
	P trend	.864	.706	.398

OR, odds ratio; CI, confidence interval

Model 1: Adjusted for sex and age(except in the age group analysis)

Model 2: Adjusted for model 1 plus diabetes duration, current smoking, current drinking, current insulin treatment, current OHA or GLP treatment, physical activity (METs), energy

Model 3: Adjusted for model 2 plus total fiber
