# Supplementary material for: Higher Iron Intake Is Independently Associated with Obesity in Younger Japanese Type-2 Diabetes Mellitus Patients
Source: Nutrients. 2022 Jan 4;14(1):211. doi: 10.3390/nu14010211 (PMC8747092; doi:10.3390/nu14010211)
Supplement: Supplementary file 1 [file nutrients-14-00211-s001.zip › Supplementary Table S4.pdf]

Supplementary Table S4. Stratified binary regression analysis of quartiles of Fe intake and obesity adjusted by nutrients.

Variable	Fe intake (quartile)	Model 1 OR (CI)	Model 2 OR (CI)	Model 3 OR (CI)
Age group ≤65 yr (n=922)	Q1 (low)	Reference	Reference	Reference
	Q2	.713 (.495-1.026)	.638 (.419-.972)	.729 (.469-1.134)
	Q3	.901 (.616-1.316)	.781 (.478-1.276)	1.015 (.588-1.753)
	Q4 (high)	.874 (.598-1.276)	.705 (.373-1.333)	1.051 (.504-2.190)
	P trend	.336	.217	.267
>65 yr (n=645)	Q1 (low)	Reference	Reference	Reference
	Q2	.936 (.593-1.476)	.742 (.443-1.245)	.898 (.522-1.543)
	Q3	.932 (.593-1.465)	.704 (.393-1.260)	.982 (.515-1.871)
	Q4 (high)	.755 (.483-1.179)	.546 (.263-1.135)	1.011 (.419-2.442)
	P trend	.622	.447	.963
Gender Female (n=579)	Q1 (low)	Reference	Reference	Reference
	Q2	.930 (.578-1.495)	.855 (.498-1.468)	.921 (.526-1.611)
	Q3	.971 (.610-1.546)	1.008 (.547-1.857)	1.195 (.606-2.356)
	Q4 (high)	.727 (.449-1.178)	.838 (.375-1.870)	1.125 (.445-2.846)
	P trend	.764	.858	.817
Male (n=988)	Q1 (low)	Reference	Reference	Reference
	Q2	.717 (.502-1.024)	.588 (.392-.881)	.743 (.484-1.141)
	Q3	.879 (.606-1.274)	.631 (.394-1.012)	.919 (.546-1.546)
	Q4 (high)	.869 (.609-1.239)	.540 (.298-.978)	1.028 (.511-2.070)
	P trend	.339	.069	.341

OR, odds ratio; CI, confidence interval

Model 1: Adjusted for age and sex(except in the gender analysis)

Model 2: Adjusted for model 1 plus diabetes duration, current smoking, current drinking, current insulin treatment, current OHA or GLP treatment, physical activity (METs), energy and macronutrients (fat, protein and carbohydrate)

Model 3: Adjusted for model 2 plus total fiber
