# Supplementary material for: Higher Iron Intake Is Independently Associated with Obesity in Younger Japanese Type-2 Diabetes Mellitus Patients
Source: Nutrients. 2022 Jan 4;14(1):211. doi: 10.3390/nu14010211 (PMC8747092; doi:10.3390/nu14010211)
Supplement: Supplementary file 1 [file nutrients-14-00211-s001.zip › Supplementary Table S5.pdf]

Supplementary Table S5. Binary regression analysis of quartiles of Fe intake and the World Health Organization (WHO) BMI cut-off for overweight(23kg/m^2) and obesity(27.5kg/m^2) for Asian population adjusted by nutrients.

Variable	Fe intake (quartile)	Model 1	Model 2	Model 3
		OR (CI)	OR (CI)	OR (CI)
BMI 23kg/m^2 All participants (n=1567)	Q1 (low)	Reference	Reference	Reference.
	Q2	.748 (.543-1.030)	.693 (.485-.992)	828 (.570-1.205)
	Q3	.859 (.619-1.192)	.782 (.518-1.180)	1.080 (.685-1.704)
	Q4 (high)	.800 (.581-1.102)	.726 (.432-1.129)	1.270 (.681-2.366)
	P trend	.322	.259	.233
Quartiles of age groups 30-54 yr (n=401)	Q1 (low)	Reference	Reference	Reference
	Q2	**.424 (.196-.914)**	.502 (.210-1.202)	.725 (.289-1.819)
	Q3	.595 (.252-1.407)	.925 (.312-2.741)	1.927 (.559-6.643)
	Q4 (high)	.793 (.326-1.931)	1.622 (.387-6.805)	5.142 (.907-29.143)
	P trend	.147	.083	**.019**
55-63 yr (n=396)	Q1 (low)	Reference	Reference	Reference
	Q2	.719 (.386-1.339)	.737 (.355-1.533)	.860 (.401-1.844)
	Q3	1.148 (.597-2.208)	1.136 (.476-2.708)	1.484 (.579-3.804)
	Q4 (high)	.628 (.339-1.166)	.667 (.225-1.981)	1.020 (.295-3.524)
	P trend	.264	.526	.564
64-71 yr (n=405)	Q1 (low)	Reference	Reference	Reference
	Q2	1.248 (.698-2.228)	.961 (.495-1.866)	.989 (.488-2.004)
	Q3	1.042 (.575-1.888)	.669 (.307-1.456)	.741 (.314-1.751)
	Q4 (high)	1.096 (.613-1.961)	.638 (.245-1.658)	.764 (.241-2.423)
	P trend	.889	.638	.856
72-89 yr (n=365)	Q1 (low)	Reference	Reference	Reference
	Q2	.630 (.329-1.206)	.569 (.276-1.174)	.748 (.352-1.589)
	Q3	.688 (.368-1.284)	.583 (.266-1.279)	1.000 (.413-2.423)

	Q4 (high)	.696 (.378-1.283)	.550 (.206-1.466)	1.487 (.431-5.129)
	P trend	.519	.464	.431
BMI 27.5kg/m^2 All participants (n=1567)	Q1 (low)	Reference	Reference	Reference
	Q2	.687 (.501-.944)	**.602 (.422-.860)**	.745 (.512-1.084)
	Q3	1.018 (.745-1.390)	.875 (.587-1.303)	1.279 (.818-1.999)
	Q4 (high)	.802 (.585-1.099)	.653 (.389-1.097)	1.220 (.664-2.242)
	P trend	.056	**.023**	**.026**
Quartiles of age groups 30-54 yr (n=401)	Q1 (low)	Reference	Reference	Reference
	Q2	.637 (.378-1.073)	**.538 (.292-.992)**	.718 (.376-1.369)
	Q3	1.280 (.723-2.265)	1.273 (.605-2.677)	2.200 (.948-5.104)
	Q4 (high)	.715 (.413-1.238)	.611 (.240-1.557)	1.368 (.460-4.070)
	P trend	.081	**.028**	**.017**
55-63 yr (n=396)	Q1 (low)	Reference	Reference	Reference
	Q2	.626 (.338-1.160)	.581 (.285-1.184)	.648 (.309-1.360)
	Q3	.911 (.515-1.612)	.733 (.341-1.575)	.887 (.384-2.050)
	Q4 (high)	1.097 (.615-1.956)	.879 (.322-2.398)	1.189 (.380-3.723)
	P trend	.392	.417	.425
64-71 yr (n=405)	Q1 (low)	Reference	Reference	Reference
	Q2	.728 (.356-1.488)	.758 (.343-1.675)	.979 (.412-2.322)
	Q3	.993 (.490-2.013)	.906 (.361-2.275)	1.422 (.503-4.021)
	Q4 (high)	.729 (.353-1.507)	.714 (.221-2.307)	1.638 (.394-6.814)
	P trend	.698	.874	.791
72-89 yr (n=365)	Q1 (low)	Reference	Reference	Reference
	Q2	.836 (.381-1.834)	.885 (.365-2.146)	1.037 (.413-2.606)
	Q3	.887 (.422-1.865)	1.100 (.422-2.863)	1.498 (.504-4.452)
	Q4 (high)	.681 (.317-1.460)	.869 (.252-2.993)	1.526 (.347-6.704)
	P trend	.794	.932	.848

OR, odds ratio; CI, confidence interval
Model 1: Adjusted for sex and age(except in the quartile of age group analysis)
Model 2: Adjusted for model 1 plus diabetes duration, current smoking, current drinking, current insulin treatment, current OHA or GLP treatment, physical activity (METs), energy and macronutrients (fat, protein and carbohydrate)
Model 3: Adjusted for model 2 plus total fiber
